# Association of Social and Behavioral Risk Factors With Earlier Onset of Adult Hypertension and Diabetes

**DOI:** 10.1001/jamanetworkopen.2019.3933

**Published:** 2019-05-17

**Authors:** Matthew S. Pantell, Aric A. Prather, Jae M. Downing, Nancy P. Gordon, Nancy E. Adler

**Affiliations:** 1 Department of Pediatrics, University of California, San Francisco; 2 Department of Psychiatry, University of California, San Francisco; 3 Department of Health Management and Policy, School of Public Health, Oregon Health & Science University, Portland; 4 Department of Health Systems & Policy, School of Public Health, Oregon Health & Science University, Portland; 5 Kaiser Permanente Division of Research, Kaiser Permanente Northern California, Oakland

## Abstract

**Importance:**

The National Academy of Medicine has recommended incorporating information on social and behavioral factors associated with health, such as educational level and exercise, into electronic health records, but questions remain about the clinical value of doing so.

**Objective:**

To examine whether National Academy of Medicine–recommended social and behavioral risk factor domains are associated with earlier onset of hypertension and/or diabetes in a clinical population.

**Design, Setting, and Participants:**

This prospective cohort study used data collected from April 1, 2005, to December 31, 2016, from a population-based sample of 41 745 patients from 4 cycles of Kaiser Permanente Northern California’s Adult Member Health Survey, administered to members at 19 Kaiser Permanente Northern California medical center service populations. The study used Kaplan-Meier survival tables and Cox proportional hazards regression analysis to estimate the onset of hypertension and diabetes among patients with no indication of disease at baseline. Data analysis was performed from June 2, 2017, to March 26, 2019.

**Exposures:**

Race/ethnicity, educational level, financial worry, partnership status, stress, intimate partner violence, concentrated neighborhood poverty, depressive symptoms, infrequent exercise, smoking, heavy alcohol consumption, and cumulative social and behavioral risk.

**Main Outcomes and Measures:**

Onset of hypertension and diabetes during the 3.5 years after survey administration.

**Results:**

The study included 18 133 people without baseline hypertension (mean [SD] age, 48.1 [15.3] years; 10 997 [60.7%] female; and 11 503 [63.4%] white) and 35 788 people without baseline diabetes (mean [SD] age, 56.2 [16.9] years; 20 191 [56.4%] female; and 24 351 [68.0%] white). There was a dose-response association between the number of social and behavioral risk factors and likelihood of onset of each condition. Controlling for age, sex, race/ethnicity, body mass index, and survey year, hazard ratios (HRs) comparing those with 3 or more risk factors with those with 0 risk factor were 1.41 (95% CI, 1.17-1.71) for developing hypertension and 1.53 (95% CI, 1.29-1.82) for developing diabetes. When the same covariates were adjusted for, having less than a high school educational level (hazard ratio [HR], 1.84; 95% CI, 1.40-2.43), being widowed (HR, 1.38; 95% CI, 1.11-1.71), concentrated neighborhood poverty (HR, 1.26; 95% CI, 1.00-1.59), infrequent exercise (HR, 1.22; 95% CI, 1.08-1.38), and smoking (HR, 1.35; 95% CI, 1.10-1.67) were significantly associated with hypertension onset. Having less than a high school educational level (HR, 1.58; 95% CI, 1.26-1.97), financial worry (HR, 1.29; 95% CI, 1.13-1.46), being single or separated (HR, 1.24; 95% CI, 1.08-1.42), high stress (HR, 1.28; 95% CI, 1.09-1.51), intimate partner violence (HR, 1.68; 95% CI, 1.14-2.48), concentrated neighborhood poverty (HR, 1.31; 95% CI, 1.07-1.60), depressive symptoms (HR, 1.28; 95% CI, 1.10-1.50), and smoking (HR, 1.53; 95% CI, 1.27-1.86) were significantly associated with diabetes onset, although heavy alcohol consumption was associated with protection (HR, 0.75; 95% CI, 0.66-0.85) rather than risk.

**Conclusions and Relevance:**

Independent of traditional risk factors, individual and cumulative social and behavioral risk factor exposures were associated with onset of hypertension and diabetes within 3.5 years in a clinical setting. The findings support the value of assessing social and behavioral risk factors to help identify high-risk patients and of providing targets for intervention.

## Key Points

**Question:**

Are social and behavioral factors associated with earlier onset of hypertension and diabetes in a clinical population?

**Findings:**

In this cohort study of 18 133 adults without baseline hypertension and 35 788 adults without baseline diabetes, cumulative social (eg, educational level) and behavioral (eg, infrequent exercise) risk was significantly associated with earlier onset of hypertension and diabetes. Those with 3 or more risk factors had the largest increased risk of developing hypertension and diabetes.

**Meaning:**

The findings suggest that social and behavioral factors associated with health should be identified in clinical settings to identify high-risk patients and inform population management.

## Teaser

This cohort study examines whether social and behavioral risk factors are associated with earlier onset of hypertension and/or diabetes among a clinical population of US adults.

## Introduction

Payment models rewarding good health outcomes rather than simply reimbursing for provision of services have created incentives for practitioners to prevent the onset of disease, slow its progression, and minimize complications.^1,2^ Recognizing the influence of social and behavioral factors on all 3 stages, health care systems are increasingly screening for social and behavioral factors associated with health. The resulting information may be used to characterize the patient population for risk stratification, enable more efficient targeting of services, and/or identify specific targets of intervention.

Health care organizations are experimenting with different ways of assessing social and behavioral risk factors. These methods differ in their breadth and focus.^3^ Health care professionals appear to assess behavioral risks, such as smoking,^4^ excessive alcohol use,^5^ and physical activity^6,7^ more commonly than unmet social needs, such as access to food^8,9^ and housing,^9^ whereas less is known about rates of screening for social risks, such as low educational level and social isolation.

The National Academy of Medicine (NAM) recommended a panel of measures that represent the key social and behavioral factors associated with health.^10^ Measures chosen were brief, psychometrically sound, and backed by empirical evidence that the domain assessed by the measure was associated with health. Subsequent studies^11,12^ found that the total panel of measures could be reliably completed, was linked to self-reported health in a community sample, and was not confounded by desirability bias (when study participants provide answers to questions that they think the researchers desire to see). However, the panel has not been tested in the context of a clinical setting to determine whether it provides clinically relevant information.

Hypertension and diabetes are 2 common conditions that put individuals at high risk of morbidity and mortality. Prevention and management are most efficient if those at highest risk can be identified in advance. We assessed whether the social and behavior risk factors identified in the NAM report are associated with the onset of hypertension and diabetes during 3.5 years of follow-up.

## Methods

Kaiser Permanente (KP) is one of the largest not-for-profit health care plans in the United States.^13^ Kaiser Permanente Northern California (KPNC) conducts an adult member health survey (MHS) every 3 years among a subset of members to assess sociodemographic characteristics. This study used data collected from April 1, 2005, to December 31, 2016, and linked survey responses from 2005, 2008, 2011, and 2014 to data in members’ electronic health records (EHRs) to determine new onset of diabetes and hypertension. Kaiser Permanente Northern California sampled members in each of 5 age groups (20-44, 45-64, 65-74, 75-79, and ≥80 years) from 19 of its largest medical centers, providing a sample varying in age, location, and racial/ethnic background. Members completed surveys by mail or online. After initial contact, they received 2 additional mailings. The survey response rate by year varied from 39.5% to 45.2%. More details about the MHS are found in the article by Gordon and Lin.^14^ This study was approved by the KPNC Institutional Review Board, which waived requirements for written consent for data collection because this was a minimal-risk study. The University of California, San Francisco (UCSF) Institutional Review Board deemed the analytic work of this study performed by UCSF researchers to be exempt. We followed the Strengthening the Reporting of Observational Studies in Epidemiology (STROBE) reporting guideline.

### Data Linkage

Data from the EHR included information from the calendar year before the survey year, the year of the survey, and the 3 years after the survey except for 2014 respondents; their EHR data were retrieved for only 2 years after the survey because of unavailability of later data at the time of abstraction. If members completed surveys in more than 1 wave, only the first survey was included. The EHR data, which were available by quarter, included months of enrollment during that quarter, clinic-measured systolic and diastolic blood pressures, body mass index (BMI) (calculated as weight in kilograms divided by height in meters squared), prescription for an antihypertensive, diagnosis of hypertension, and diagnosis of diabetes. Clinical data were available only for quarters in which members made a visit.

The final source of data was the American Community Survey (ACS), which provides neighborhood information. On the basis of member mailing addresses, data from the ACS were coded for the percentage of households in a Federal Information Processing System–defined area living at or below the federal poverty level. Staff from the KP Division of Research abstracted and linked all KP and ACS data, which were deidentified (including removing address data) before sharing with University of California, San Francisco researchers for analysis.

### Study Sample

Of the 59 220 members with available data, 1606 (2.7%) were excluded because they had missing sex, race/ethnicity, or BMI data, and an additional 6477 (11.2% of the remaining sample) were excluded because of missing social or behavioral data. In addition, 1709 patients were excluded because they had no visit (outpatient or emergency department) during the study period. Finally, to ensure that lapse in insurance status did not contribute to difference in time to diagnosis, we excluded an additional 7683 members without continuous enrollment in KPNC during the follow-up period.

Of the 41 745 eligible members remaining, we constructed 2 separate but overlapping analytic samples to study onset of hypertension and onset of diabetes. Participants excluded from hypertension analyses because of a baseline hypertension diagnosis were still eligible for the diabetes sample and vice versa. The resulting hypertension sample included 18 133 members who had none of the following within 1 year before the baseline survey: an EHR-recorded diagnosis of hypertension, a systolic blood pressure of 140 mm Hg or higher or diastolic blood pressure of 90 mm Hg or higher, a prescription filled for an antihypertensive medication in the previous year, or self-report of ever having been diagnosed with or treated for hypertension. The diabetes analytic sample included 35 788 members who had no diagnosis of diabetes or self-report of diabetes treatment in the year before baseline survey completion.

### Independent Variables

The following variables were constructed from items on the MHS (eTable 1 in the Supplement gives the exact wording): (1) race/ethnicity (black, Hispanic, Asian, white non-Hispanic, and other race/ethnicity); (2) educational level (less than high school, high school, some college, and college or more); (3) financial worry (answering yes to having worried a great deal about your or you family’s finances and financial security during the past 12 months); (4) partner status (married or partnered, widowed, and single, separated, or divorced); (5) high stress (answering most or much of the time to the question, “During the past 12 months, how often did you feel very stressed, tense, or anxious?”); (6) intimate partner violence (IPV) (answering “yes” to having been physically hurt, abused, or fearing for your safety because of anger or threats of a current or former spouse or partner or boyfriend or girlfriend during the past 12 months; this question was excluded from the second additional mailed survey in 2014); (7) concentrated neighborhood poverty (living in a Federal Information Processing System–defined area where ≥30% of households are at or below the federal poverty line); (8) depressive symptoms (reporting depression, sadness, or very low spirits lasting ≥2 weeks in the past year or taking prescription medicine for depression in the past year); (9) infrequent exercise (exercising <3 times each week); (10) smoking status (current smoker, former smoker, or never smoker); and (11) heavy alcohol consumption (using an Alcohol Use Disorders Identification Test Consumption [AUDIT-C] score cutoff of ≥4 for men and ≥3 for women; although the cutoff for risk of problems from alcohol is usually 4 for men and 3 for women,^15^ the MHS only has 2 of the 3 AUDIT-C questions, making a total available score of 8 instead of 12).

Finally, we constructed a cumulative social and behavioral risk variable. Social risk factors often operate together rather than in isolation,^16^ and summations of social risk factors have been used to study a range of health outcomes.^17,18,19,20^ We therefore assigned 1 point for each of the following: less than a college education, financial worry, being unpartnered, high stress, experiencing or fearing IPV, living in a neighborhood of concentrated poverty, depressive symptoms, infrequent exercise, currently smoking, and heavy alcohol consumption. The resulting score ranged from 0 to 10. Three or more risk factors were collapsed into 1 category (≥3), allowing for similar sample sizes in each risk group.

### Outcomes

 Outcomes were new diagnosis of hypertension or diabetes noted in the EHR during any quarter after the quarter in which the survey was completed.

### Covariates

Because of their associations with health, social, and behavioral characteristics,^21,22,23,24^ the following were included as covariates in analyses: age (continuous), sex (woman, man), race/ethnicity, and BMI (normal or underweight [<24], overweight [25 to <30], or obese [≥30]).

### Statistical Analysis

All analyses were performed using Stata statistical software, version 15.0 (StataCorp). Kaplan-Meier survival tables were constructed to compare onset of hypertension and diabetes based on the cumulative risk score, and log-rank test statistics were calculated for equality based on the sum of risks, with a cutoff of 2-tailed *P* = .05. Cox proportional hazards regression models were used to assess the risk of onset of each outcome in separate models that controlled for age, sex, race/ethnicity, BMI, and a survey year indicator variable using only 1 social and behavioral risk factor domain or the cumulative risk score in each analysis and a cutoff of 2-tailed *P* = .05 to indicate significance.

We additionally performed several sensitivity analyses. Because practitioners may lack complete social and behavioral data, we computed a Cox proportional hazards regression analysis using the same cumulative social and behavioral risk variable, but instead of excluding members with missing social and behavioral risk factor data, we assigned each missing domain a 0. This approach mimics when practitioners and health care systems have information on only some social and behavioral risk factor domains.

To investigate whether social and behavioral factors were associated with the presence or absence of disease after 3 years (vs the rate of onset), we also performed logistic regressions that mirrored the Cox proportional hazards regression models that we performed. These analyses excluded 2014 members (n = 4862) because they had potentially 2.5 instead of 3.5 years of available follow-up data. Using the sample with no missing data, we also compared receiver operator characteristic curves and the area under the curve (AUC) of models with only covariates to models that also included the cumulative social and behavioral risk variable to assess disease onset of hypertension and diabetes. We used χ^2^ testing to compare the AUC between the 2 models for hypertension and diabetes, using *P* = .05 as a cutoff for significance. Data analysis was performed from June 2, 2017, to March 26, 2019.

## Results

The study included 18 133 people without baseline hypertension (mean [SD] age, 48.1 [15.3] years; 10 997 [60.7%] female; and 11 503 [63.4%] white) and 35 788 people without baseline diabetes (mean [SD] age, 56.2 [16.9] years; 20 191 [56.4%] female; and 24 351 [68.0%] white). Sociodemographic characteristics of each analytic sample are given in Table 1. In the hypertension sample, 8931 (49.3%) were overweight or obese, and the prevalence of social and behavioral risk factors ranged from a low of 1.6% for IPV to a 10 high of 47.7% for less than a college education. In the diabetes sample, 58.3% were overweight or obese, and prevalence of risk factors ranged from 1.3% for IPV to 54.4% for less than a college education. There were similar distributions by year and social risk in each analytic sample.

**Table 1.** Population Characteristics of Each Sample Analyzed^a^

Characteristic	Patients Without Hypertension (n = 18 133)	Patients Without Diabetes (n = 35 788)
Age, mean (SD), y	48.1 (15.3)	56.2 (16.9)
Sex		
Male	7136 (39.4)	15 597 (43.6)
Female	10 997 (60.7)	20 191 (56.4)
BMI		
<25	9202 (50.8)	14 958 (41.8)
25 to <30	6043 (33.3)	13 008 (36.4)
≥30	2888 (15.9)	7822 (21.9)
Race/ethnicity		
Asian	3218 (17.8)	5157 (14.4)
Hispanic	2109 (11.6)	3372 (9.4)
Black	829 (4.6)	2018 (5.6)
White	11 503 (63.4)	24 351 (68.0)
Other	474 (2.6)	890 (2.5)
Educational level		
Less than high school	381 (2.1)	1268 (3.5)
High school degree	2256 (12.4)	5837 (16.3)
Some college	6005 (33.1)	12 368 (34.6)
College or more	9491 (52.3)	16 315 (45.6)
Financial worry	4480 (24.7)	7882 (22.0)
Partner status		
Single or separated	3395 (18.7)	6408 (17.9)
Widowed	578 (3.2)	2701 (7.6)
Married or partnered	14 160 (78.1)	26 679 (74.6)
High stress	2874 (15.9)	4803 (13.4)
Intimate partner violence	293 (1.6)	477 (1.3)
Concentrated neighborhood poverty	912 (5.0)	1 895 (5.3)
Depressive symptoms	2105 (11.6)	4 203 (11.7)
Infrequent exercise	6209 (34.2)	12 231 (34.2)
Smoking status		
Current	1329 (7.3)	2515 (7.0)
Former	3940 (21.7)	10 323 (28.8)
Never	12 864 (70.9)	22 950 (64.1)
Heavy drinking	6195 (34.2)	12 202 (34.1)
No. of social and behavioral risks		
0	3154 (17.4)	5846 (16.3)
1	6059 (33.4)	11 749 (32.8)
2	4534 (25.0)	9219 (25.8)
≥3	4386 (24.2)	8974 (25.1)
Survey year		
2005	5523 (30.5)	10 834 (30.3)
2008	4937 (27.2)	9828 (27.5)
2011	5566 (30.7)	11 018 (30.8)
2014	2107 (11.6)	4108 (11.5)

Abbreviation: BMI, body mass index (calculated as weight in kilograms divided by height in meters squared).

^a^ Data are presented as number (percentage) of patients unless otherwise indicated. All percentages are rounded to the nearest tenth; thus, some percentages may total more than 100.

**Figure 1.**
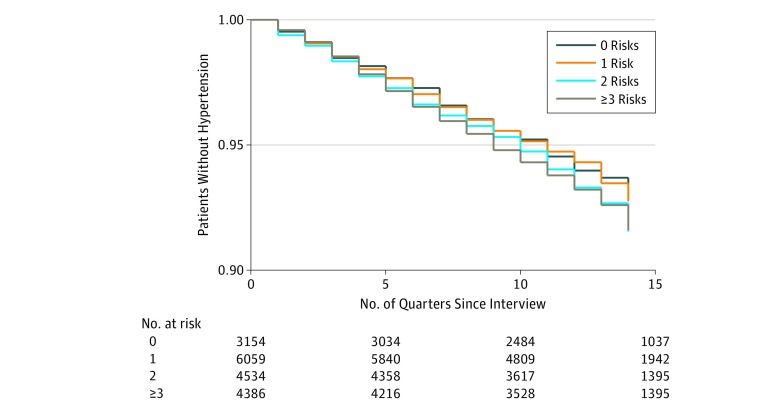
Kaplan-Meier Survival Estimates for Patients Without Hypertension

**Figure 2.**
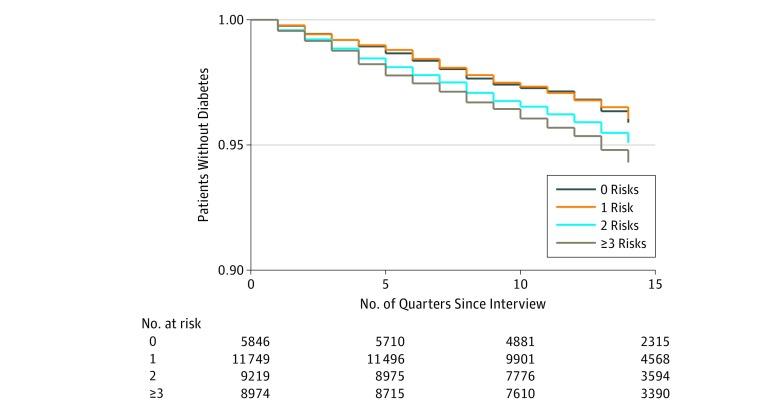
Kaplan-Meier Survival Estimates for Patients Without Diabetes

Overall, 1163 patients (6.4%) in the eligible sample developed hypertension by the end of the follow-up period, ranging from 183 (5.8%) of those with no social or behavioral risk factors to 307 (7.0%) of those with 3 or more. Of those without baseline diabetes, 1442 (4.0%) developed diabetes, ranging from 203 patients (3.5%) with no social or behavioral risk factors to 445 patients (5.0%) with 3 or more risks factors. Figure 1 and Figure 2 show the Kaplan-Meier plots of incidence of hypertension and diabetes by cumulative risk. Patterns were generally similar by sex (eTable 2 and eTable 3 in the Supplement); thus, results were pooled.

Besides other race/ethnicity, all nonwhite racial/ethnic groups were at increased risk of developing hypertension compared with white individuals (Table 2). In addition, having a lower educational level (hazard ratio [HR] comparing less than high school with college or more, 1.84; 95% CI, 1.40-2.43), being widowed (HR, 1.38; 95% CI 1.11-1.71), concentrated neighborhood poverty (HR, 1.26; 95% CI, 1.00-1.59), infrequent exercise (HR, 1.22; 95% CI, 1.08-1.38), and smoking currently (HR, 1.35; 95% CI, 1.10-1.67) were associated with earlier hypertension onset.

**Table 2.** Risk of Developing Hypertension and Diabetes by Individual Social and Behavioral Factors^a^

Factor	Patients Without Hypertension (n = 18 133)		Patients Without Diabetes (n = 35 788)	
	HR (95% CI)	*P* Value	HR (95% CI)	*P* Value
Race/ethnicity				
Asian	1.55 (1.31-1.84)	<.001	2.31 (1.98-2.69)	<.001
Hispanic	1.28 (1.05-1.56)	.01	1.46 (1.22-1.74)	<.001
Black	1.50 (1.16-1.92)	.002	1.27 (1.03-1.57)	.02
White	1 [Reference]	NA	1 [Reference]	NA
Other	1.20 (0.84-1.72)	.32	1.57 (1.17-2.12)	.003
Educational level				
Less than high school	1.84 (1.40-2.43)	<.001	1.58 (1.26-1.97)	<.001
High school	1.53 (1.29-1.81)	<.001	1.47 (1.27-1.70)	<.001
Some college	1.40 (1.23-1.60)	<.001	1.39 (1.22-1.58)	<.001
College or more	1 [Reference]	NA	1 [Reference]	NA
Financial worry	1.07 (0.92-1.23)	.39	1.29 (1.13-1.46)	<.001
Partner status				
Widowed	1.38 (1.11-1.71)	.004	1.10 (0.93-1.31)	.27
Single or separated	1.14 (0.98-1.33)	.09	1.24 (1.08-1.42)	.002
Partnered	1 [Reference]	NA	1 [Reference]	NA
High stress	1.11 (0.93-1.33)	.26	1.28 (1.09-1.51)	.003
Intimate partner violence	1.02 (0.64-1.62)	.94	1.68 (1.14-2.48)	.009
Concentrated neighborhood poverty	1.26 (1.00-1.59)	.049	1.31 (1.07-1.60)	.008
Depressive symptoms	1.05 (0.87-1.26)	.63	1.28 (1.10-1.50)	.001
Infrequent exercise	1.22 (1.08-1.38)	.002	1.35 (1.21-1.50)	<.001
Smoking status				
Current	1.35 (1.10-1.67)	.005	1.53 (1.26-1.86)	<.001
Former	1.07 (0.93-1.22)	.35	1.08 (0.96-1.21)	.20
Never	1 [Reference]	NA	1 [Reference]	NA
Heavy drinking	0.97 (0.86-1.10)	.65	0.75 (0.66-0.85)	<.001
No. of risk factors				
0	1 [Reference]	NA	1 [Reference]	NA
1	1.05 (87-1.25)	.63	1.02 (0.86-1.20)	.86
2	1.24 (1.03-1.50)	.02	1.25 (1.05-1.49)	.01
≥3	1.41 (1.17-1.71)	<.001	1.53 (1.29-1.82)	<.001

Abbreviations: HR, hazard ratio; NA, not applicable.

^a^ Model covariates were age, sex, race/ethnicity, body mass index, and survey year.

All nonwhite racial/ethnic groups were at significantly higher risk of developing diabetes than white individuals (Table 2). Each social and behavioral domain was independently significantly associated with diabetes onset. Heavy alcohol consumption had a negative association with diabetes onset (HR, 0.75; 95% CI, 0.66-0.85).

With the Cox proportional hazards regression models for cumulative social and behavioral risks, both outcomes exhibited a graded association between onset of disease and cumulative social risk—the higher the number of risk factors, the higher the risk of developing disease. Those with 3 or more risks had an HR of 1.41 (95% CI, 1.17-1.71; *P* < .001) for developing hypertension and an HR of 1.53 (95% CI, 1.29-1.82; *P* < .001) for developing diabetes.

The sensitivity analyses found similar overall patterns, including an association between an increasing number of social and behaviors risk factors and increased likelihood of disease (eTables 4-6 in the Supplement). However, Asian and Hispanic race/ethnicity were not significantly associated with hypertension onset and black race was not associated with diabetes onset when using logistic regression (eTable 5 in the Supplement).

The AUC of the logistic regression models without missing data estimating hypertension using only baseline covariates was 0.762 (95% CI, 0.748-0.776), which was similar to the AUC of the model that included the cumulative social and behavioral risk variable AUC of 0.765 (95% CI, 0.751-0.779), although these estimates were significantly different from each other (*P* = .02). Similarly, the AUC of the baseline logistic regression model estimating diabetes was 0.746 (95% CI, 0.734-0.758), which was similar to but significantly less than the AUC of 0.751 (95% CI, 0.739-0.762) for the model with the cumulative social and behavioral risk factor (*P* = .001).

## Discussion

Since the release of the NAM report^10^ recommending a panel of social and behavioral risk factor measures to be incorporated into all EHRs, a number of practitioners have begun screening their patients. A wide range of health care professionals and systems are grappling with whether to screen for social and behavioral risk factors, for which social and behavioral risk factors they should screen, and whom they should screen. Supporting the value of using the NAM measures in a clinical setting, the findings of the current study provide relevant evidence on each of those questions. With regard to whether to screen, the results are consistent with prior work showing an association of social and behavioral risk factors and the incidence of hypertension and diabetes^20^ and add to the literature on achieving more precise predictive risk modeling by combining social and clinical information.^25^ In the samples of patients with no evidence of hypertension or diabetes at baseline, social and behavioral risk factor measures were associated with disease onset for a relatively short period. This finding suggests that social and behavioral risk factor information could help practitioners better target resources to patients who most need more intensive preventive care. Although the added power of social and behavioral risk factors for identifying earlier onset of hypertension and diabetes in an individual may appear to be small, even a modest increase in cumulative incidence of diseases associated with social and behavioral risk factors could inform population health management, helping to identify groups of patients at higher risk of disease onset.

The results support the value of screening for a range of NAM-endorsed social and behavioral risk factors. Although most individual social and behavioral risk factor measures were significantly associated with disease onset, some were not. Knowing which social and behavioral risk factors affect a patient can guide referrals to specific interventions. However, patients with higher overall social and behavioral risk factor scores were at greater risk of disease onset regardless of which specific social and behavioral risk factors were reported, suggesting the value of broad screening. Patients with higher overall social and behavioral burden may warrant closer follow-up for disease surveillance than those with a similar clinical profile but few total social and behavioral risks.

Finally, the study informs the question of who should be screened. The association of social and behavioral risk factors with disease risk was found among a sample of patients in KPNC, who represent a diverse insured population in the United States. This finding challenges the assumption that screening for social and behavioral risk factors is unnecessary outside safety-net settings.

As more practitioners screen for social and behavioral risk factors, it will be increasingly important to gain consensus on standard measures with evidence-based cut points, which would expedite data sharing across facilities to improve clinical care and facilitate development and evaluation of predictive models. The recent release of a social and behavioral risk factor module in Epic, a popular EHR system, should help systematize collection of social and behavioral risk factors. The Epic module encompasses NAM measures and adds selected social needs, such as food and housing insecurity.^26^

The adoption of EHRs has permitted access to an ever-expanding amount of data and aided the development of precision medicine. As interventions become increasingly individualized, it will be important to include social and behavioral risk factors in addition to traditional biometric data. These domains may increase the precision of not only estimating disease onset but also choosing relevant interventions, which is important to help achieve high-value care in a system with finite resources.

### Limitations

Limitations of our study should be noted. Although the sample of patients enrolled in KPNC is diverse, it is not nationally representative. The sample in our study was also relatively small and consisted of an insured population. Previous analyses have found that compared with the Northern California non-KP population, the KPNC population has a lower proportion of Hispanic women and a lower proportion of people with lower income, lower educational levels, and part-time employment.^27^ Taken together, this limits the generalizability of our findings. In addition, because we were limited to 2.5 to 3.5 years of follow-up, we cannot know whether associations differ during longer periods.

In addition, the social and behavioral risk factor measures in the MHS overlap with, but are not identical to, those recommended in the NAM report.^10^ The MHS measures address similar domains, but some are worded differently and/or use fewer items. For example, the MHS includes a subset of the AUDIT-C scale, whereas the NAM measure of alcohol consumption includes the full scale. This difference could have been associated with a decreased sensitivity for identifying heavy drinkers and might explain the finding that heavy use of alcohol had a protective association with diabetes onset. To the extent that MHS measures are sparser and less reliable, the findings may underestimate the associations on the NAM scores with disease onset.

## Conclusions

The current study underscores the potential importance of knowing social and behavioral information in both clinical and health care system settings and advocates for exploring the utility of routine collection of this information. There will be logistical challenges to accomplishing this,^28^ but our findings support the potential value of doing so.

## Notes

Supplement. **eTable 1.** Member Health Survey (MHS) Questions From Which Domains Were Derived **eTable 2.** Risk of Developing Hypertension and Diabetes by Social and Behavioral Factors, Among Women **eTable 3.** Risk of Developing Hypertension and Diabetes by Social and Behavioral Factors, Among Men **eTable 4.** Risk of Developing Hypertension and Diabetes by Social and Behavioral Factors, Including Those with Missing Social and Behavioral Data **eTable 5.** Odds of Developing Hypertension and Diabetes by Social and Behavioral Factors **eTable 6.** Odds of Developing Hypertension and Diabetes by Social and Behavioral Factors, Including Those with Missing Social and Behavioral Data
