# Supplementary material for: Association of Social and Behavioral Risk Factors With Earlier Onset of Adult Hypertension and Diabetes
Source: JAMA Netw Open. 2019 May 17;2(5):e193933. doi: 10.1001/jamanetworkopen.2019.3933 (PMC6537925; doi:10.1001/jamanetworkopen.2019.3933)
Supplement: Supplement. — eTable 1. Member Health Survey (MHS) Questions From Which Domains Were Derived eTable 2. Risk of Developing Hypertension and Diabetes by Social and Behavioral Factors, Among Women eTable 3. Risk of Developing Hypertension and Diabetes by Social and Behavioral Factors, Among Men eTable 4. Risk of Developing Hypertension and Diabetes by Social and Behavioral Factors, Including Those with Missing Social and Behavioral Data eTable 5. Odds of Developing Hypertension and Diabetes by Social and Behavioral Factors eTable 6. Odds of Developing Hypertension and Diabetes by Social and Behavioral Factors, Including Those with Missing Social and Behavioral Data [file jamanetwopen-2-e193933-s001.pdf]

Supplementary Online Content

Pantell MS, Prather AA, Downing JM, Gordon NP, Adler NE. Association of social and behavioral risk factors with earlier onset of adult hypertension and diabetes. *JAMA Netw Open*. 2019;2(5):e193933. doi:10.1001/jamanetworkopen.2019.3933

eTable 1. Member Health Survey (MHS) Questions From Which Domains Were Derived

eTable 2. Risk of Developing Hypertension and Diabetes by Social and Behavioral Factors, Among Women

eTable 3. Risk of Developing Hypertension and Diabetes by Social and Behavioral Factors, Among Men

eTable 4. Risk of Developing Hypertension and Diabetes by Social and Behavioral Factors, Including Those with Missing Social and Behavioral Data

eTable 5. Odds of Developing Hypertension and Diabetes by Social and Behavioral Factors

eTable 6. Odds of Developing Hypertension and Diabetes by Social and Behavioral Factors, Including Those with Missing Social and Behavioral Data

This supplementary material has been provided by the authors to give readers additional information about their work.

© 2019 Pantell MS et al. *JAMA Network Open.*

**eTable 1.** Member Health Survey (MHS) Questions from Which Domains Were Derived		
Social or Behavioral Domain	MHS Question(s)	MHS Answer Options
Race/ethnicity	Describe your race & ethnicity	White or Euro-American; African-American; Other Black; Mexican or Central American; Other Hispanic/Latino; Other Hispanic/Latino (Specified; or written in); Middle Eastern; North African or Central Asian (e.g.; Iranian); So Asian (Indian; Pakistani; Afghani; etc.); Chinese; Filipino; Japanese; Korean; Southeast Asian; Southeast Asian (Specified; or written in); Other Asian; Other Asian (Specified; or written in); Hawaiian/Pacific Islander; Hawaiian/Pacific Islander (Specified or written in); Native Amer. Indian/Alaska Native; Other (Specify)
Education	Highest level school completed?	8th grade or less (primary or middle); 9th-11th grade (some high school); 12th grade (High School grad or G.E.D.); Tech/Trade School Certificate; Some college (no degree); Associate's Degree (AA; AS; etc); Bachelor's Degree (BA; BS; etc); Graduate/professional degree (MA; MD; etc)
Financial worry	During the past 12 mos., any of these situations or problems occur? You worried a great deal about your/family's finances/financial security	Yes; no
Partner status	What is your current marital status? (Check only ONE)	Married; in a committed relationship; widowed; single, divorced, or separated
High stress	During the past 12 months, how often did you feel very stressed, tense or anxious?	Most of the time; much of the time; some of the time; a little of time; never
Intimate partner violence	During the past 12 mos., any of these situations or problems occur? You were physically hurt, abused or feared for your safety because of anger or threats of a current or former spouse/partner or boyfriend/girlfriend[a]	Yes; no
Concentrated neighborhood poverty	N/a	N/a
Depressive symptoms	During the past 12 months, how often did you feel depressed or sad?	Most of the time; much of the time; some of the time; a little of time; never
Infrequent exercise	How often do you usually get physical exercise?	5 or > times/week; 3-4 times/week; 1-2 times/week; 2-4 times/month; once a month or less; never
Smoking status	Have you ever regularly smoked cigarettes daily (for at least 1 year)?	Yes; no; never smoked
	Do you smoke cigarettes now, even occasionally?	Yes; no
Heavy alcohol consumption	During the past 12 months, how often had a drink containing alcohol?	Almost every day; 5 to 6 times/week; 3 to 4 times/week; 1 or 2 times/week; 2-4 times/month; 1 time a month or less; never in past 12 mos (used to drink); never at all (never drank as adult)
	How many drinks did you usually have?	1-20; "few" or "some"; "lot", "many", or "several"
a For 2014, questionnaires to members age 65 and older added "or a person you depend on for care"		

© 2019 Pantell MS et al. *JAMA Network Open.*

**eTable 2.** Risk of Developing Hypertension and Diabetes by Social and Behavioral Factors, Among Women												
		Hypertension (N = 10,997)						Diabetes (N = 20,191)				
		HR	CI			*P*-Value		HR	CI			*P*-Value
Race/Ethnicity												
	Other Race/Ethnicity	1.26	0.75	-	2.12	0.38		1.95	1.30	-	2.93	0.001
	Asian	1.66	1.31	-	2.10	<0.001		2.46	1.97	-	3.06	<0.001
	Hispanic	1.32	1.01	-	1.73	0.04		1.57	1.21	-	2.02	0.001
	Black	1.70	1.24	-	2.33	<0.001		1.41	1.07	-	1.86	0.01
	White	Ref	-		-			Ref	-		-	
Education												
	<HS	1.93	1.29	-	2.90	0.002		1.41	1.00	-	1.99	0.049
	High School	1.51	1.19	-	1.91	0.001		1.47	1.19	-	1.81	<0.001
	Some College	1.39	1.16	-	1.67	<0.001		1.24	1.02	-	1.49	0.03
	College +	Ref	-		-			Ref	-		-	
Financial Worry		1.05	0.84	-	1.31	0.66		1.45	1.22	-	1.72	<0.001
Partner Status												
	Widowed	1.31	1.01	-	1.71	0.04		1.06	0.86	-	1.32	0.58
	Single/Sep	1.08	0.88	-	1.33	0.44		1.14	0.94	-	1.38	0.17
	Partnered	Ref	-		-			Ref	-		-	
High Stress		1.05	0.85	-	1.29	0.68		1.29	1.04	-	1.59	0.02
Intimate Partner Violence		1.23	0.72	-	2.09	0.45		1.91	1.19	-	3.06	0.01
Concentrated Neighborhood Poverty		1.13	0.82	-	1.57	0.45		1.44	1.09	-	1.89	0.01
Depressive Symptoms		0.98	0.78	-	1.23	0.87		1.22	1.00	-	1.50	0.049
Infrequent Exercise		1.20	1.01	-	1.42	0.04		1.35	1.16	-	1.57	<0.001
Smoking Status												
	Current	1.14	0.83	-	1.57	0.42		1.69	1.27	-	2.25	<0.001
	Former	0.99	0.81	-	1.19	0.89		1.19	1.00	-	1.41	0.048
	Never	Ref	-		-			Ref	-		-	
Heavy Drinking		0.92	0.78	-	1.10	0.38		0.70	0.58	-	0.84	<0.001
No. Risk Factors[a]												
	3+	1.27	0.96	-	1.68	0.10		1.62	1.22	-	2.16	0.001
	2	1.09	0.81	-	1.45	0.58		1.27	0.95	-	1.71	0.11
	1	1.04	0.78	-	1.38	0.80		1.10	0.81	-	1.49	0.53
	0	Ref	-		-			Ref	-		-	

[a] For this analysis, if a member had missing data for a social or behavioral variable, that variable was set to "0" when constructing the sum of all social and behavioral risk factors.

© 2019 Pantell MS et al. *JAMA Network Open.*

Abbreviations: HR, hazard ratio; CI, confidence interval.
Model covariates: Age, sex, race/ethnicity, BMI, and survey year.

eTable 3. Risk of Developing Hypertension and Diabetes by Social and Behavioral Factors, Among Men

	Hypertension (N = 7,136)					Diabetes (N = 15,597)				
	HR	CI			*P*-Value	HR	CI			*P*-Value
Race/Ethnicity										
Other Race/Ethnicity	1.16	0.70	-	1.92	0.56	1.27	0.82	-	1.97	0.28
Asian	1.44	1.12	-	1.85	0.005	2.18	1.76	-	2.70	0.000
Hispanic	1.26	0.94	-	1.69	0.12	1.36	1.06	-	1.75	0.02
Black	1.20	0.78	-	1.83	0.40	1.15	0.84	-	1.59	0.38
White	Ref	-		-		Ref	-		-	
Education										
<HS	1.77	1.21	-	2.59	0.003	1.71	1.27	-	2.29	<0.001
High School	1.55	1.21	-	1.98	<0.001	1.43	1.16	-	1.76	0.001
Some College	1.42	1.17	-	1.71	<0.001	1.53	1.29	-	1.81	<0.001
College +	Ref	-		-		Ref	-		-	
Financial Worry	1.06	0.84	-	1.33	0.63	1.13	0.94	-	1.37	0.20
Partner Status										
Widowed	1.49	0.97	-	2.28	0.07	1.09	0.80	-	1.48	0.60
Single/Sep	1.19	0.94	-	1.50	0.15	1.37	1.12	-	1.67	0.002
Partnered	Ref	-		-		Ref	-		-	
High Stress	1.21	0.89	-	1.66	0.23	1.31	1.01	-	1.70	0.05
Intimate Partner Violence	0.63	0.23	-	1.67	0.35	1.35	0.67	-	2.70	0.40
Concentrated Neighborhood Poverty	1.39	1.00	-	1.94	0.05	1.18	0.88	-	1.58	0.28
Depressive Symptoms	1.16	0.85	-	1.58	0.36	1.39	1.10	-	1.76	0.01
Infrequent Exercise	1.24	1.04	-	1.49	0.02	1.34	1.16	-	1.56	<0.001
Smoking Status										
Current	1.57	1.18	-	2.09	0.002	1.42	1.09	-	1.84	0.009
Former	1.16	0.96	-	1.40	0.12	1.01	0.86	-	1.18	0.92
Never	Ref	-		-		Ref	-		-	
Heavy Drinking	1.04	0.87	-	1.25	0.65	0.79	0.67	-	0.94	0.01
No. Risk Factors[a]										
3+	1.54	1.18	-	2.01	0.001	1.46	1.17	-	1.83	0.001
2	1.39	1.09	-	1.78	0.01	1.25	1.01	-	1.55	0.04
1	1.03	0.82	-	1.30	0.80	0.97	0.79	-	1.19	0.76

© 2019 Pantell MS et al. *JAMA Network Open.*

	0	Ref	-		-							

[a] For this analysis, if a member had missing data for a social or behavioral variable, that variable was set to "0" when constructing the sum of all social and behavioral risk factors.

Abbreviations: HR, hazard ratio; CI, confidence interval.

Model covariates: Age, sex, race/ethnicity, BMI, and survey year.

© 2019 Pantell MS et al. *JAMA Network Open.*

eTable 4. Risk of Developing Hypertension and Diabetes by Social and Behavioral Factors, Including Those with Missing Social and Behavioral Data

		Hypertension (N = 19,976)						Diabetes (N = 39,941)				
		HR	CI			*P*-Value		HR	CI			*P*-Value
No. Risk Factors[a]												
	3+	1.33	1.11	-	1.59	0.002		1.49	1.28	-	1.74	<0.001
	2	1.22	1.03	-	1.44	0.02		1.15	0.99	-	1.33	0.06
	1	1.06	0.91	-	1.23	0.49		1.01	0.88	-	1.15	0.94
	0	Ref	-		-			Ref	-		-	

[a] For this analysis, if a member had missing data for a social or behavioral variable, that variable was set to "0" when constructing the sum of all social and behavioral risk factors.

Abbreviations: HR, hazard ratio; CI, confidence interval.

Model covariates: Age, sex, race/ethnicity, BMI, and survey year.

© 2019 Pantell MS et al. *JAMA Network Open.*

eTable 5. Odds of Developing Hypertension and Diabetes by Social and Behavioral Factors

	Hypertension (N = 16,026)					Diabetes (N = 31,680)				
	OR	CI			*P*-Value	OR	CI			*P*-Value
Race/Ethnicity										
Other Race/Ethnicity	1.57	0.96	-	2.56	0.07	2.04	1.36	-	3.07	0.001
Asian	1.18	0.93	-	1.50	0.17	1.59	1.27	-	1.99	<0.001
Hispanic	1.22	0.93	-	1.61	0.15	1.39	1.08	-	1.80	0.01
Black	2.01	1.42	-	2.85	<0.001	1.19	0.88	-	1.60	0.26
White	Ref	-		-		Ref	-		-	
Education										
<HS	1.98	1.43	-	2.73	<0.001	1.62	1.27	-	2.07	<0.001
High School	1.60	1.33	-	1.93	<0.001	1.49	1.27	-	1.74	<0.001
Some College	1.47	1.27	-	1.70	<0.001	1.45	1.27	-	1.66	<0.001
College +	Ref	-		-		Ref	-		-	
Financial Worry	1.12	0.96	-	1.32	0.16	1.34	1.17	-	1.54	<0.001
Partner Status										
Widowed	1.41	1.09	-	1.81	0.01	1.14	0.94	-	1.37	0.18
Single/Sep	1.18	1.00	-	1.39	0.05	1.27	1.09	-	1.47	0.002
Partnered	Ref	-		-		Ref	-		-	
High Stress	1.10	0.90	-	1.35	0.33	1.29	1.08	-	1.55	0.006
Intimate Partner Violence	1.02	0.61	-	1.71	0.93	1.74	1.14	-	2.64	0.009
Concentrated Neighborhood Poverty	1.31	1.01	-	1.69	0.043	1.37	1.10	-	1.69	0.004
Depressive Symptoms	1.06	0.87	-	1.30	0.56	1.34	1.13	-	1.58	0.001
Infrequent Exercise	1.25	1.09	-	1.43	0.002	1.40	1.24	-	1.57	<0.001
Smoking Status										
Current	1.40	1.11	-	1.76	0.004	1.55	1.26	-	1.91	<0.001
Former	1.10	0.95	-	1.28	0.19	1.07	0.95	-	1.22	0.27
Never	Ref	-		-		Ref	-		-	
Heavy Drinking	0.98	0.86	-	1.13	0.83	0.75	0.66	-	0.86	<0.001
No. Risk Factors										
3+	1.51	1.22	-	1.86	<0.001	1.65	1.36	-	1.99	<0.001
2	1.32	1.07	-	1.62	0.008	1.31	1.09	-	1.58	0.005
1	1.08	0.88	-	1.31	0.47	1.05	0.87	-	1.26	0.62

© 2019 Pantell MS et al. *JAMA Network Open.*

	0	Ref	-		-			Ref	-		-	

Abbreviations: OR, odds ratio; CI, confidence interval.

Model covariates: Age, sex, race/ethnicity, BMI, and survey year.

© 2019 Pantell MS et al. *JAMA Network Open.*

eTable 6. Odds of Developing Hypertension and Diabetes by Social and Behavioral Factors, Including Those with Missing Social and Behavioral Data

		Hypertension (N = 17,068)						Diabetes (N = 34,424)				
		OR	CI			*P*-Value		OR	CI			*P*-Value
No. Risk Factors[a]												
	3+	1.44	1.18	-	1.76	<0.001		1.62	1.36	-	1.92	<0.001
	2	1.27	1.06	-	1.53	0.01		1.19	1.01	-	1.40	0.04
	1	1.10	0.93	-	1.31	0.26		1.04	0.89	-	1.21	0.64
	0	Ref	-		-			Ref	-		-	

[a] For this analysis, if a member had missing data for a social or behavioral variable, that variable was set to "0" when constructing the sum of all social and behavioral risk factors.

Abbreviations: OR, odds ratio; CI, confidence interval.

Model covariates: Age, sex, race/ethnicity, BMI, and survey year.

© 2019 Pantell MS et al. *JAMA Network Open.*
